# Carrier frequency and incidence estimation of familial hemophagocytic lymphohistiocytosis in East Asian populations by genome aggregation database (gnomAD) based analysis

**DOI:** 10.3389/fped.2022.975665

**Published:** 2022-11-11

**Authors:** Jong Eun Park, Taeheon Lee, Kyeongsu Ha, Eun Hye Cho, Chang-Seok Ki

**Affiliations:** ^1^ Department of Laboratory Medicine, Hanyang University Guri Hospital, Hanyang University College of Medicine, Guri, South Korea; ^2^ GC Genome, Yongin, South Korea; ^3^ Department of Laboratory Medicine, Kangbuk Samsung Hospital, Sungkyunkwan University School of Medicine, Seoul, South Korea

**Keywords:** familial hemophagocytic lymphohistiocytosis, gnomAD, carrier frequency, East Asian, Korean

## Abstract

**Objectives:**

Hemophagocytic lymphohistiocytosis (HLH) is a clinical syndrome characterized by a life-threatening condition caused by severe hypercytokinemia. The hereditary forms of HLH, also called familial HLH (fHLH), have 4 different genes (*PRF1, UNC13D, STX11,* and *STXBP2*) and have been identified as being causative for fHLH. This study aimed to analyze the carrier frequency and expected incidence of fHLH in East Asians and Koreans using exome data from the Genome Aggregation Database (gnomAD).

**Methods:**

We analyzed 9,197 exomes for East Asian populations from gnomAD with 1,909 Korean for four fHLH genes. All identified variants were classified according to 2015 American College of Medical Genetics and Genomics and the Association for Molecular Pathology guideline.

**Results:**

19 pathogenic variant/likely pathogenic variants (PV/LPVs) were identified in 30 East Asian individuals (0.33%). Among them, 7 PV/LPVs were identified in 17 Korean individuals (0.63%). The estimated incidence of fHLH was 1 in 1,105,652 for East Asians and l in 235,128 for Koreans.

**Conclusions:**

This study is the first to identify carrier frequencies in East Asian and Korean populations for fHLH using gnomAD. It was confirmed that the carrier frequency of fHLH patients was high in Koreans among East Asians and the incidence was also predicted to be higher than that of other East Asians. The variant spectrum of fHLH genes in East Asian and Korean populations differed greatly from those of other ethnic groups.

## Introduction

Hemophagocytic lymphohistiocytosis (HLH) is a clinical syndrome characterized by a life-threatening condition caused by severe hypercytokinemia due to an uncontrolled immune response (1, 2). HLH may occur as a hereditary or acquired condition. The hereditary form of HLH, also called familial HLH (fHLH), was first described in 1952 (3). Since the *PRF1* gene was first discovered as a causative gene of fHLH in 1999 (4), 4 different genes (*PRF1, UNC13D, STX11,* and *STXBP2*) to date have been identified as being causative for fHLH (5). These genes are inherited as autosomal recessive and involved in lymphocyte granule-dependent cytotoxicity (6). In addition, genetic defects for pigmentary disorders, X-linked lymphoproliferative syndromes, and EBV susceptibility disorders are associated with HLH (7).

Although the incidence of HLH has been known to be 0.34–1.8 per 100,000 births, few studies exist with regard to the incidence of HLH (8, 9). Also, carrier frequencies for the four fHLH major genes (*PRF1, UNC13D, STX11,* and *STXBP2*) are unknown to our knowledge except for *PRF1* (10). According to a recent study, there is a study result that the prognosis can be improved when preemptive allogeneic hematopoietic stem cell transplantation is performed in asymptomatic pediatric patients with pathogenic billeleic variants (11). Therefore, it would be important to identify carrier frequencies and use them to determine the expected incidence.

The Genome Aggregation Database (gnomAD) is a widely used genomic database around the world. The gnomAD V2 is consists of 125,748 exomes and 4,359 genomes (12). The gnomAD V2 contains exome data from 9,197 East Asians, including 1,909 Koreans. Among the open genome databases, it contains the most data on East Asians and Koreans, making it suitable for research on East Asians and Koreans. The four genes (*PRF1, UNC13D, STX11,* and *STXBP2*) variants interpreted according to the 2015 American College of Medical Genetics and Genomics and the Association for Molecular Pathology guidelines (2015 ACMG/AMP guidelines), which are currently generally adopted in clinical practice (13). This study aimed to analyze the carrier frequency and expected incidence of fHLH in East Asians and Koreans using exome data from the gnomAD through 2015 ACMG/AMP guidelines.

## Materials and methods

### GnomAD East Asian population data

The gnomAD data (v2.1.1) for the *PRF1, UNC13D, STX11,* and *STXBP2* genes were obtained from gnomAD (https://gnomad.broadinstitute.org/). We analyzed 9,197 East Asian exomes of which 1,909 were from Koreans, 76 were from Japanese, and 7,212 were from other East Asian populations. The filtered variants that were flagged in gnomAD as failing “InbreedingCoeff,” “AC0,” or “RF” QC filters were excluded from the analysis.

### Variant classification and statistical analysis

All variants were interpreted using 2015 ACMG/AMP guidelines and Sequence Variant Interpretation (SVI) general recommendations for ACMG/AMP Criteria by ClinGen (https://clinicalgenome.org/working-groups/sequence-variant-interpretation/, accessed on 1 November, 2021). The 2015 ACMG/AMP guidelines recommend the classification of variants into five categories: pathogenic variants (PV), likely pathogenic variants (LPV), variants of uncertain significance, likely benign variants, and benign variants. REVEL (14), CADD (15), Polyphen-2 (16), SIFT (17) and SpliceAI (18) were used to predict variant pathogenicity. Variants identified from 9,197 East Asian exomes in gnomAD were compared with previously classified disease-causing variants from the Human Gene Mutation Database (HGMD) and ClinVar, which are representative disease databases, to identify overlap. The HGMD professional database (http://www.hgmd.org/, release 2018.03) is a comprehensive collection of germline variants categorized into six categories, of which we analyzed only disease-causing mutations (DM). ClinVar (https://www.ncbi.nlm.nih.gov/clinvar/, accessed on 16 October 2021) is a freely available archive that provides the classification of variants interpreted by clinical laboratories.

### fHLH carrier frequency and incidence estimation

East Asian and Korean carrier frequencies were calculated for fHLH genes (*PRF1, UNC13D, STX11,* and *STXBP2)* using gnomAD. We used those classified as PV and LPV according to the 2015 ACMG/AMP guidelines, the disease-causing variant (DM) in HGMD and those classified as PV and LPV in ClinVar for carrier frequency analysis. Thereafter, we estimated the incidence of fHLH based on frequency and the Hardy–Weinberg equilibrium principle (1 = *p*^2^ + 2*pq* + *q*^2^). The detailed prediction method has been described in a previous study (19). We then summed the estimated carrier frequencies and incidence across all genes. MedCalc ver. 11.5.1.0 (MedCalc Software, Maiakerke, Belgium) was used for statistical analysis and 95% confidence intervals (CIs) were calculated for each value.

## Results

9,197 East Asian exomes including 1,909 Korean exomes were analyzed for *PRF1, UNC13D, STX11,* and *STXBP2* gene variants. These variants were classified according to 2015 ACMG/AMP guidelines and two disease classification databases, HGMD and ClinVar (Table 1). According to the 2015 ACMG/AMP guidelines, 19 PV/LPVs were identified in 30 East Asian individuals (30/9,191 = 0.33%). Among them, 7 PV/LPVs were identified in 17 Korean individuals (12/1,909 = 0.63%). The estimated incidence of fHLH was 1 in 1,105,652 in East Asians and l in 235,128 in Koreans. Based on HGMD, the carrier frequency was 6.61% in East Asians and 4.61% in Koreans. Estimated incidence values were 1 in 1,129 for East Asians and l in 2,856 for Koreans. Based on ClinVar, the carrier frequency was 0.27% in East Asians and 0.73% in Koreans. Estimated incidences were 1 in 1,138,896 for East Asians and l in 137,502 for Koreans.

**Table 1:** Carrier frequency and estimated incidence of familial hemophagocytic lymphohistiocytosis syndrome in east Asian and Korean populations.

	Variants (*n*)	Total individuals (*n*)	Carrier frequency (%), (95% CI)	Estimated incidence (1/*n*), (95% CI)
gnomAD East Asian exomes (*n* = 9,197)				
2015 ACMG/AMP (PV/LPV)	19	30	0.33 (0.22–0.47)	1/1,105,652 (1/327,863 - 1/4,327,486)
HGMD (DM)	30	608	6.61 (6.10–7.16)	1/1,129 (1/950 - 1/1,344)
ClinVar (PV/LPV)	11	25	0.27 (0.18–0.40)	1/1,138,896 (1/363,956 - 1/3,871,353)
gnomAD Korean exomes (*n* = 1,909)				
2015 ACMG/AMP (PV/LPV)	7	12	0.63 (0.32–1.1)	1/235,128 (1/41,544 - 1/1,949,236)
HGMD (DM)	17	88	4.61 (3.70–5.68)	1/2,856 (1/1,719 - 1/4,802)
ClinVar (PV/LPV)	6	14	0.73 (0.40–1.23)	1/137,502 (1/32,082 - 1/744,763)

2015 ACMG/AMP, 2015 American College of Medical Genetics and Genomics and the Association for Molecular Pathology guideline; 95% CI, 95% confidence intervals; DM, disease-causing variant; gnomAD, Genome Aggregation Database; LPV, likely pathogenic variant; PV, pathogenic variant.

PV/LPVs found in East Asians and Koreans in the four fHLH genes are summarized in Table 2 and Supplementary Table S1. A total of 30 alleles were identified in East Asians, among them 14 alleles (14/30, 46.7%) were the *PRF1* gene, 9 alleles (9/30, 30.0%) were the *UNC13D* gene, 2 alleles (2/30, 6.7%) were the *STX11* gene, and 5 alleles (5/30, 16.7%) were the *STXBP2* gene. In Koreans, 6 alleles (6/12, 50.0%) were the *PFR1* gene, 5 alleles (5/12, 41.7%) were the *UNC13D* gene, and 1 allele (1/12, 8.3%) was the *STXBP2* gene. Variants classified as DM in HGMD and variants classified as PV/LPV in ClinVar are summarized in Supplementary Tables S2 and S3.

**Table 2:** Pathogenic variants and likely pathogenic variants of East Asian and Korean populations in gnomAD.

Gene	Nucleotide change	Amino acid change	gnomAD allele frequency (allele count / allele number)									Functional study
			Korean	East Asian	African	Latino	Ashkenazi Jewish	European (Finnish)	European (non-Finnish)	Other	South Asian	
			(*n* = 1,909)	(*n* = 9,197)	(*n* = 8,128)	(*n* = 17,296)	(*n* = 5,040)	(*n* = 10,824)	(*n* = 56,885)	(*n* = 3,070)	(*n* = 15,308)	
*PRF1*	c.65del	p.Pro22ArgfsTer29	0.0005241 (2/3,816)	0.0001675 (3/17,912)	0	0	0	0	0	0	0	(20, 21)
*PRF1*	c.16°C > T	p.Arg54Cys	0	0.000109 (2/18,342)	0	0.000116 (4/34,472)	0	0	0.000008925 (1/112,048)	0	0.00006559 (2/30,494)	(22)
*PRF1*	c.673C > T	p.Arg225Trp	0	0.00005439 (1/18,386)	0	0	0	0	0.00001781 (2/112,300)	0	0	(4, 22, 23)
*PRF1*	c.1066C > T	p.Arg356Trp	0	0.00005459 (1/18,320)	0	0	0	0	0.00001785 (2/112,044)	0	0	(24)
*PRF1*	c.1090_1091del	p.Leu364GlufsTer93	0.0007862 (3/3,816)	0.0001642 (3/18,274)	0	0	0	0	0	0	0	(20, 21)
*PRF1*	c.1168C > T	p.Arg390Ter	0.0002625 (1/3,810)	0.00005471 (1/18,278)	0	0	0	0	0	0	0	(25)
*PRF1*	c.1244C > G	p.Ala415Gly	0	0.000109 (2/18,346)	0	0	0	0	0	0	0	-
*PRF1*	c.1349C > T	p.Thr450Met	0	0.00005437 (1/18,392)	0	0	0	0	0.000008812 (1/113,486)	0	0.0001633 (5/30,616)	(23, 24, 26)
*UNC13D*	c.247C > T	p.Arg83Ter	0.0002619 (1/3,818)	0.0000545 (1/18,348)	0	0	0	0	0	0	0	-
*UNC13D*	c.754-1G > C		0.0007874 (3/3,810)	0.0001634 (3/18,364)	0	0	0	0	0	0	0	(27)
*UNC13D*	c.1055 + 1G > A		0.0002619 (1/3,818)	0.0000544 (1/18,382)	0	0	0	0.00004637 (1/21,566)	0	0	0	(28)
*UNC13D*	c.1358del	p.Pro453HisfsTer6	0	0.0000544 (1/18,384)	0	0	0	0	0	0	0	-
*UNC13D*	c.2054_2055del	p.Ser685PhefsTer19	0	0.0000544 (1/18,382)	0	0	0	0	0	0	0	-
*UNC13D*	c.2972del	p.Pro991ArgfsTer38	0	0.00005621 (1/17,792)	0	0	0	0	0	0	0	-
*UNC13D*	c.3193C > T	p.Arg1065Ter	0	0.00007113 (1/14,058)	0	0.00003545 (1/28,212)	0	0	0	0	0	(29)
*STX11*	c.59del	p.Pro20GlnfsTer43	0	0.00005438 (1/18,390)	0	0	0	0	0	0	0	-
*STX11*	c.496G > T	p.Glu166Ter	0	0.00005452 (1/18,342)	0	0	0	0	0	0	0	-
*STXBP2*	c.577A > C	p.Lys193Gln	0.0004766 (1/2,098)	0.00008693 (1/11,504)	0	0	0	0	0.00001627 (1/61,452)	0	0	(30)
*STXBP2*	c.1214G > A	p.Arg405Gln	0	0.0002181 (4/18,342)	0	0	0	0	0	0	0	-

gnomAD, Genome Aggregation Database.

Carrier frequency for each fHLH gene was *PRF1* 0.15%, *UNC13D* 0.10%, *STXBP2* 0.05%, and *STX11* 0.02% in East Asians, while it was *PRF1* 0.31%, *UNC13D* 0.26%, *STXBP2* 0.05%, and *STX11* 0% in Koreans (Supplementary Tables S4–7). In East Asians, the estimated incidence was 1/1,726,221 for *PRF1*, 1/ 4,176,857 for *UNC13D*, 1/84,555,423 for *STX11*, and 1/13,531,356 for *STXBP2*. In Koreans, the estimated incidence was 1/404,920 for *PRF1*, 1/583,162 for *UNC13D*, 1/3,641,979 for *STX11*, and 1/14,579,043 for *STXBP2*.

*STXBP2* c.1214G > A was the most common variant in East Asians but was not found in other ethnicities including Koreans and Japanese. *PRF1* c.1090_1091del and *UNC13D* c.754-1G > A variants were the most common in Koreans. These variants were not identified in other ethnicities and were the only variants found in Koreans. In addition, variants other than *PRF1* c.1168C > T, *UNC13D* c.1055 + 1G > A, and *UNC13D* c.247C > T were found only in Koreans.

## Discussion

In this study, the carrier frequency and estimated incidence of fHLH was analyzed for East Asians and Koreans using gnomAD. The carrier frequency of East Asians was 0.33% and the carrier frequency of Koreans was 0.63%. However, it was difficult to compare because carrier frequency studies of fHLH have not been actively performed; with regard to in practice resources for carrier screening published in ACMG, the maximal carrier frequency of *PRF1* was described as 0.67% (10). In this study, the *PRF1* carrier frequency was 0.15% in East Asians and 0.31% in Koreans. The carrier frequency of *PRF1* was lower than in a prior study. Within East Asians, it was found that the carrier frequency of fHLH was higher in Koreans than in other East Asians.

Through this study, we were able to estimate the approximate incidence of fHLH. When calculated by the Hardy-Weinberg equation, the incidence of fHLH in East Asians was predicted to be 1/1,105,652 (0.09 per 100,000 births) and 1/235,128 (0.43 per 100,000 births) in Koreans. This was lower than the incidence reported in Sweden (1.8 per 100,000 births) and higher than that in Japan (0.34 per 100,000 births), one of the East Asian populations (8, 9). However, since this study was analyzed with exome data registered in gnomAD, the *UNC13D* c.118–308C > T variant, known as the Korean founder variant, was not included in this study because it is located in the deep intron (31). According to the Korean Reference Genome Database (KRGDB, http://152.99.75.168:9090/KRGDB/menuPages/introKor.jsp), the *UNC13D* c.118–308C > T variant was identified as a minor allele frequency of 0.001364 in Koreans. Therefore, the actual incidence in East Asians, especially Koreans, was estimated to be higher.

In this study, the fHLH gene carrier frequencies were *PRF1* (0.15%), *UNC13D* (0.10%), *STXBP2* (0.05%), and *STX11* (0.02%) in the order of East Asians and *PRF1* (0.31%), *UNC13D* (0.26%), *STXBP2* (0.05%), and *STX11* (0%) in Koreans. The distribution of genes in fHLH patients was also estimated to be similar to that of carriers. According to a large-scale study of 1,892 HLH patients, variants were found in the order of *PRF1, STXBP2, UNC13D*, and *STX11* among 197 genetically confirmed patients (32). Among East Asians, *UNC13D, PRF1,* and *STXBP2* were found in Chinese and *PRF1, UNC13D,* and *STXBP2* were found in Japanese in that order (33, 34). In Koreans, *UNC13D* was found more frequently than *PRF1* (31). In this study, *PRF1* was found to be slightly more frequent than *UNC13D*; if *UNC13D* c.118–308C > T, which was not included in this study, was included, it could be estimated that *UNC13D* was similar to *PRF1*.

The PV/LPVs identified in this study were found to have a significantly different variant spectrum from other ethnicities. In gnomAD, c.1090_1091del in the *PRF1* gene and c.754–1G > A in the *UNC13D* gene was mainly observed in Koreans; however, this variant was not found in other ethnicities. In addition, variants found in Koreans were only found in Koreans, except for *PRF1* c.65del, *UNC13D* c.1055 + 1G > A, and *STXBP2* c.1214G > A. All variants identified in East Asians were not found in Ashkenazi Jews or Africans and some *PRF1* variants were found in Europeans (non-Finnish). In the largest study of the genetic analysis of HLH patients, c.50del and c.445G > A in the *PRF1* gene and c.143 °C > T and c.1247-1G > C in the *STXBP2* gene, known to be frequently reported in HLH patients, were not found in East Asians in gnomAD (32). Even among East Asians, Koreans had a different variant spectrum (Supplementary Figure S1). Among the 19 variant fHLH genes found in Koreans, all variants except for the c.65del variant of the *PRF1* gene were unique to Koreans. From this, it could be inferred that the variant spectrum of fHLH genes differed between East Asians and other ethnicities, and that Koreans are unique even within East Asians.

When the variants found in Korean fHLH patients were compared with those of Korean carriers identified in gnomAD, it was confirmed that the *PRF1* and *UNC13D* variants found in this study were all major variants in Korean HLH patients (31, 35). *PRF1* c.1090_1091del was the variant with the highest carrier frequency in Koreans in gnomAD and was found most frequently in HLH patients where the *PRF1* variant was confirmed. The c.118-308C > T variant in *UNC13D* could not be confirmed in this study; however, the c.754-1G > A, c.1055 + 1G > A, and c.247C > T variants found in *UNC13D* were all found in Korean fHLH patients. The c.754-1G > A variant, which was the most frequent in *UNC13D* was also found in patients. Through the results of this study, it was confirmed that predicting the carrier in gnomAD was helpful toward predicting the genetic spectrum in actual patients.

According to data from the Korean Statistical Information Service (http://kosis.kr/; accessed on 26 January 2022) in 2022, the total population of Korea was 51.8 million with 272,337 births. Based on the carrier frequency in this study, the number of carriers is estimated to be 0.3 million with 1,716 in newborns per year. The estimated incidence of fHLH in Korea based on the Hardy–Weinberg equilibrium is 2.7 cases per year.

With the recent development of sequencing technology, studies applying genomic sequencing to healthy or ill newborns have been reported (36, 37). These studies classified categories according to clinical actionability and the age of onset and evaluated whether genomic sequencing would be useful for diseases for which early diagnosis would be beneficial. In one of the representative studies of the BabySeq Project, all four fHLH genes were classified as category A (genes included in the newborn genomic sequencing report with definitive or strong evidence to cause a highly penetrant childhood-onset disorder) (38). In another representative study, the North Carolina Newborn Exome Sequencing for Universal Screening, only *UNC13D* was classified as category 1 (pediatric onset conditions with high actionability); the rest of the genes were not included in other categories (39). In order to confirm the cost-effectiveness of genomic newborn screening, it would be important to check the carrier frequency and incidence of each disease and gene. In that respect, this study predicting the carrier frequency and incidence of fHLH is considered to be a significant study.

This study has some limitations. We did not analyze structural variations including the large deletion/insertion of the fHLH gene. We also couldn't include the deep intron variant. The c.118-308C > T variant of *UNC13D* is known to be common in Korean fHLH patients but was not included in this study, it is thus expected that the carrier frequency in Koreans may be underestimated. We identified only four fHLH-related genes. However, in addition to fHLH, there are other HLH predisposition genes such as granule/pigment abnormality genes and X-linked lymphoproliferative disease genes, so further analysis is needed. Nonetheless, this study makes several valuable contributions. This is the largest study among those performed in East Asia with regard to the analysis of four fHLH genes. To the best of our knowledge, there have been no large-scale population studies of carrier frequencies and the estimated fHLH incidence in Koreans. We believe that this study more accurately predicted the carrier frequency of fHLH in East Asians and Koreans. In addition, this study was a meaningful study that could confirm that public data analysis is helpful toward predicting the variant spectrum of actual patients.

## Conclusions

This study is the first to identify carrier frequencies in East Asians and Koreans for fHLH using gnomAD. We confirmed that the carrier frequency of fHLH patients was high in Koreans among East Asians and the incidence was also predicted to be higher than in other East Asian populations. The variant spectrum of fHLH genes in East Asian and Korean populations differed greatly from those of other ethnic groups. Our data are expected to serve as a reference for further investigations of fHLH in East Asian and Korean populations.

## Data Availability

The original contributions presented in the study are included in the article/**supplementary materials**, further inquiries can be directed to the corresponding authors.
