# Supplementary material for: Carrier frequency and incidence estimation of familial hemophagocytic lymphohistiocytosis in East Asian populations by genome aggregation database (gnomAD) based analysis
Source: Front Pediatr. 2022 Nov 11;10:975665. doi: 10.3389/fped.2022.975665 (PMC9692074; doi:10.3389/fped.2022.975665)
Supplement: Supplementary file 1 [file Datasheet1.pdf]

Supplementary Table 1. In-silico analysis of Pathogenic variants and likely pathogenic variants of East Asian in gnomAD population

Gene	Nucleotide change	Amino acid change	gnomAD allele frequency		in-silico analysis			
			Korean	East Asain	REVEL	CADD	Polyphen-2	SIFT
PRF1	c.65del	p.Pro22ArgfsTer29	0.0005241	0.0001675	NA	NA	NA	NA
PRF1	c.160C>T	p.Arg54Cys	0	0.000109	0.545	19.47	0.996	0.01
PRF1	c.673C>T	p.Arg225Trp	0	0.00005439	0.583	23.9	0.999	0.01
PRF1	c.1066C>T	p.Arg356Trp	0	0.00005459	0.5299	16.10	0.208	0.06
PRF1	c.1090_1091del	p.Leu364GlufsTer93	0.0007862	0.0001642	NA	NA	NA	NA
PRF1	c.1168C>T	p.Arg390Ter	0.0002625	0.00005471	NA	35	NA	NA
PRF1	c.1244C>G	p.Ala415Gly	0	0.000109	0.5749	23.2	0.446	0.42
PRF1	c.1349C>T	p.Thr450Met	0	0.00005437	0.8679	24.7	0.999	0
UNC13D	c.247C>T	p.Arg83Ter	0.0002619	0.0000545	NA	36	NA	NA
UNC13D	c.754-1G>C	.	0.0007874	0.0001634	NA	NA	NA	NA
UNC13D	c.1055+1G>A	.	0.0002619	0.0000544	NA	NA	NA	NA
UNC13D	c.1358del	p.Pro453HisfsTer6	0	0.0000544	NA	NA	NA	NA
UNC13D	c.2054_2055del	p.Ser685PhefsTer19	0	0.0000544	NA	NA	NA	NA
UNC13D	c.2972del	p.Pro991ArgfsTer38	0	0.00005621	NA	NA	NA	NA
UNC13D	c.3193C>T	p.Arg1065Ter	0	0.00007113	NA	53	NA	NA
STX11	c.59del	p.Pro20GlnfsTer43	0	0.00005438	NA	NA	NA	NA
STX11	c.496G>T	p.Glu166Ter	0	0.00005452	NA	40	NA	NA
STXBP2	c.577A>C	p.Lys193Gln	0.0004766	0.00008693	0.194	22.2	0.009	0.23
STXBP2	c.1214G>A	p.Arg405Gln	0	0.0002181	0.7409	32	1.000	0

Abbreviations: gnomAD, Genome Aggregation Database; NA, not applicable.

Supplementary Table 2. Disease-causing mutations in the Human Gene Mutation Database of East Asian and Korean populations in gnomAD.

Gene	Nucleotide change	Amino acid change	gnomAD allele frequency		2015 ACMG/AMP
			Korean	East Asian	
PRF1	c.46C>T	p.Pro16Ser	0	0.00005836	VUS
PRF1	c.65del	p.Pro22ArgfsTer29	0.0005241	0.0001675	LPV
PRF1	c.112G>A	p.Val38Met	0	0.00005466	VUS
PRF1	c.148G>C	p.Val50Leu	0	0.0006562	VUS
PRF1	c.160C>T	p.Arg54Cys	0	0.000109	LPV
PRF1	c.503G>A	p.Ser168Asn	0	0.0003806	VUS
PRF1	c.563C>T	p.Pro188Leu	0	0.00005437	VUS
PRF1	c.673C>T	p.Arg225Trp	0	0.00005439	LPV
PRF1	c.674G>A	p.Arg225Gln	0.0005238	0.0004351	VUS
PRF1	c.1042G>A	p.Val348Met	0.0002619	0.0001089	VUS
PRF1	c.1066C>T	p.Arg356Trp	0	0.00005459	LPV
PRF1	c.1090_1091del	p.Leu364GlufsTer93	0.0007862	0.0001642	PV
PRF1	c.1168C>T	p.Arg390Ter	0.0002625	0.00005471	PV
PRF1	c.1228C>T	p.Arg410Trp	0.00131	0.0003818	LPV
PRF1	c.1349C>T	p.Thr450Met	0	0.00005437	LPV
PRF1	c.1465A>T	p.Arg489Trp	0	0.00005437	VUS
UNC13D	c.247C>T	p.Arg83Ter	0.0002619	0.0000545	LPV
UNC13D	c.754-1G>C	.	0.0007874	0.0001634	LPV
UNC13D	c.1055+1G>A	.	0.0002619	0.0000544	LPV
UNC13D	c.1228A>C	p.Ile410Leu	0.01074	0.01588	VUS
UNC13D	c.1232G>A	p.Arg411Gln	0.0005238	0.007832	VUS
UNC13D	c.1760G>A	p.Arg587His	0	0.0001127	VUS

UNC13D	c.2588G>A	p.Gly863Asp	0.002881	0.003644	VUS
UNC13D	c.3134C>T	p.Thr1045Met	0.001931	0.001075	VUS
UNC13D	c.3193C>T	p.Arg1065Ter	0	0.00007113	LPV
UNC13D	c.3229_3235del	p.Arg1077SerfsTer48	0.00173	0.001275	VUS
STX11	c.842T>G	p.Phe281Cys	0.0005238	0.0002741	VUS
STXBP2	c.577A>C	p.Lys193Gln	0.0004766	0.00008693	LPV
STXBP2	c.971A>G	p.Lys324Arg	0.0002619	0.00005437	VUS
STXBP2	c.1214G>A	p.Arg405Gln	0	0.0002181	LPV

Abbreviations: 2015 ACMG/AMP, 2015 American College of Medical Genetics and Genomics and the Association for Molecular Pathology guideline; gnomAD, Genome Aggregation Database; LPV, likely pathogenic variant; PV, pathogenic variant; VUS, variant of uncertain significance.

Supplementary Table 3. Pathogenic variants and likely pathogenic variants in ClinVar of East Asian and Korean populations in gnomAD.

Gene	Nucleotide change	Amino acid change	gnomAD allele frequency		2015 ACMG/AMP
			Korean	East Asian	
PRF1	c.160C>T	p.Arg54Cys	0	0.000109	LPV
PRF1	c.673C>T	p.Arg225Trp	0	0.00005439	LPV
PRF1	c.1066C>T	p.Arg356Trp	0	0.00005459	LPV
PRF1	c.1090_1091del	p.Leu364GlufsTer93	0.0007862	0.0001642	PV
PRF1	c.1168C>T	p.Arg390Ter	0.0002625	0.00005471	PV
PRF1	c.1228C>T	p.Arg410Trp	0.00131	0.0003818	LPV
PRF1	c.1349C>T	p.Thr450Met	0	0.00005437	LPV
UNC13D	c.247C>T	p.Arg83Ter	0.0002619	0.0000545	LPV
UNC13D	c.754-1G>C	.	0.0007874	0.0001634	LPV
UNC13D	c.1055+1G>A	.	0.0002619	0.0000544	LPV
STXBP2	c.1214G>A	p.Arg405Gln	0	0.0002181	LPV

Abbreviations: 2015 ACMG/AMP, 2015 American College of Medical Genetics and Genomics and the Association for Molecular Pathology guideline; gnomAD, Genome Aggregation Database; LPV, likely pathogenic variant; PV, pathogenic variant; VUS, variant of uncertain significance.

Supplementary Table 4. Carrier frequency and estimated incidence of *PRF1* genes in East Asian and Korean populations.

	Variants (n)	Total individuals (n)	Carrier frequency (%), (95% CI)	Estimated incidence (1/n), (95% CI)
gnomAD East Asian exomes (n=9,197)				
2015 ACMG/AMP (PV/LPV)	8	14	0.15 (0.08 - 0.26)	1/1,726,221 (1/613,223 - 1/5,778,476)
HGMD (DM)	16	52	0.57 (0.42 - 0.74)	1/125,126 (1/72,770 - 1/224,294)
ClinVar (PV/LPV)	7	16	0.17 (0.10 - 0.28)	1/1,321,178 (1/501,214 - 1/4,048,435)
gnomAD Korean exomes (n=1,909)				
2015 ACMG/AMP (PV/LPV)	3	6	0.31 (0.12 - 0.68)	1/404,920 (1/85,471 - 1/3,008,856)
HGMD (DM)	6	14	0.73 (0.40 - 1.23)	1/74,367 (1/26,418 - 1/248,879)
ClinVar (PV/LPV)	3	9	0.47 (0.22 - 0.90)	1/179,927 (1/49,936 - 1/860,523)

Abbreviations: 2015 ACMG/AMP, 2015 American College of Medical Genetics and Genomics and the Association for Molecular Pathology guideline; 95% CI, 95% confidence intervals; DM, disease-causing variant; gnomAD, Genome Aggregation Database; LPV, likely pathogenic variant; PV, pathogenic variant.

Supplementary Table 5. Carrier frequency and estimated incidence of *UNC13D* genes in East Asian and Korean populations.

	Variants (n)	Total individuals (n)	Carrier frequency (%), (95% CI)	Estimated incidence (1/n), (95% CI)
gnomAD East Asian exomes (n=9,197)				
2015 ACMG/AMP (PV/LPV)	7	9	0.1 (0.04 - 0.19)	1/4,176,857 (1/1,159,193 - 1/19,974,408)
HGMD (DM)	10	545	5.93 (5.44 - 6.45)	1/1,139 (1/963 - 1/1,352)
ClinVar (PV/LPV)	3	5	0.05 (0.02 - 0.13)	1/13,531,356 (1/2,485,090 - 1/128,401,640)
gnomAD Korean exomes (n=1,909)				
2015 ACMG/AMP (PV/LPV)	3	5	0.26 (0.09 - 0.61)	1/583,162 (1/107,076 - 1/5,536,332)
HGMD (DM)	8	70	3.67 (2.86 - 4.63)	1/2,975 (1/1,864 - 1/4,897)
ClinVar (PV/LPV)	3	5	0.26 (0.09 - 0.61)	1/583,162 (1/107,076 - 1/5,536,332)

Abbreviations: 2015 ACMG/AMP, 2015 American College of Medical Genetics and Genomics and the Association for Molecular Pathology guideline; 95% CI, 95% confidence intervals; DM, disease-causing variant; gnomAD, Genome Aggregation Database; LPV, likely pathogenic variant; PV, pathogenic variant.

Supplementary Table 6. Carrier frequency and estimated incidence of *STX11* genes in East Asian and Korean populations.

	Variants (n)	Total individuals (n)	Carrier frequency (%), (95% CI)	Estimated incidence (1/n), (95% CI)
gnomAD East Asian exomes (n=9,197)				
2015 ACMG/AMP (PV/LPV)	2	2	0.02 (0 - 0.08)	1/84,555,423 (1/6,482,874 - 1/5,782,937,443)
HGMD (DM)	1	5	0.05 (0.02 - 0.13)	1/13,531,356 (1/2,485,090 - 1/128,401,640)
ClinVar (PV/LPV)	0	0	0 (0 - 0.04)	-
gnomAD Korean exomes (n=1,909)				
2015 ACMG/AMP (PV/LPV)	0	0	0 (0 - 0.19)	-
HGMD (DM)	1	2	0.10 (0.01 - 0.38)	1/3,641,979 (1/279,208 - 1/248,000,496)
ClinVar (PV/LPV)	0	0	0 (0 - 0.19)	-

Abbreviations: 2015 ACMG/AMP, 2015 American College of Medical Genetics and Genomics and the Association for Molecular Pathology guideline; 95% CI, 95% confidence intervals; DM, disease-causing variant; gnomAD, Genome Aggregation Database; LPV, likely pathogenic variant; PV, pathogenic variant.

Supplementary Table 7. Carrier frequency and estimated incidence of *STXBP2* genes in East Asian and Korean populations.

	Variants (n)	Total individuals (n)	Carrier frequency (%), (95% CI)	Estimated incidence (1/n), (95% CI)
gnomAD East Asian exomes (n=9,197)				
2015 ACMG/AMP (PV/LPV)	2	5	0.05 (0.02 - 0.13)	1/13,531,356 (1/2,485,090 - 1/128,401,640)
HGMD (DM)	3	6	0.07 (0.02 - 0.14)	1/9,397,927 (1/1,983,733 - 1/69,792,973)
ClinVar (PV/LPV)	1	4	0.04 (0.01 - 0.11)	1/21,168,043 (1/3,225,533 - 1/284,854,635)
gnomAD Korean exomes (n=1,909)				
2015 ACMG/AMP (PV/LPV)	1	1	0.05 (0 - 0.29)	1/14,579,043 (1/469,581 - 1/22,612,923,286)
HGMD (DM)	2	2	0.1 (0.01 - 0.38)	1/3,641,979 (1/279,208 - 1/248,000,496)
ClinVar (PV/LPV)	0	0	0 (0 - 0.19)	-

Abbreviations: 2015 ACMG/AMP, 2015 American College of Medical Genetics and Genomics and the Association for Molecular Pathology guideline; 95% CI, 95% confidence intervals; DM, disease-causing variant; gnomAD, Genome Aggregation Database; LPV, likely pathogenic variant; PV, pathogenic variant.

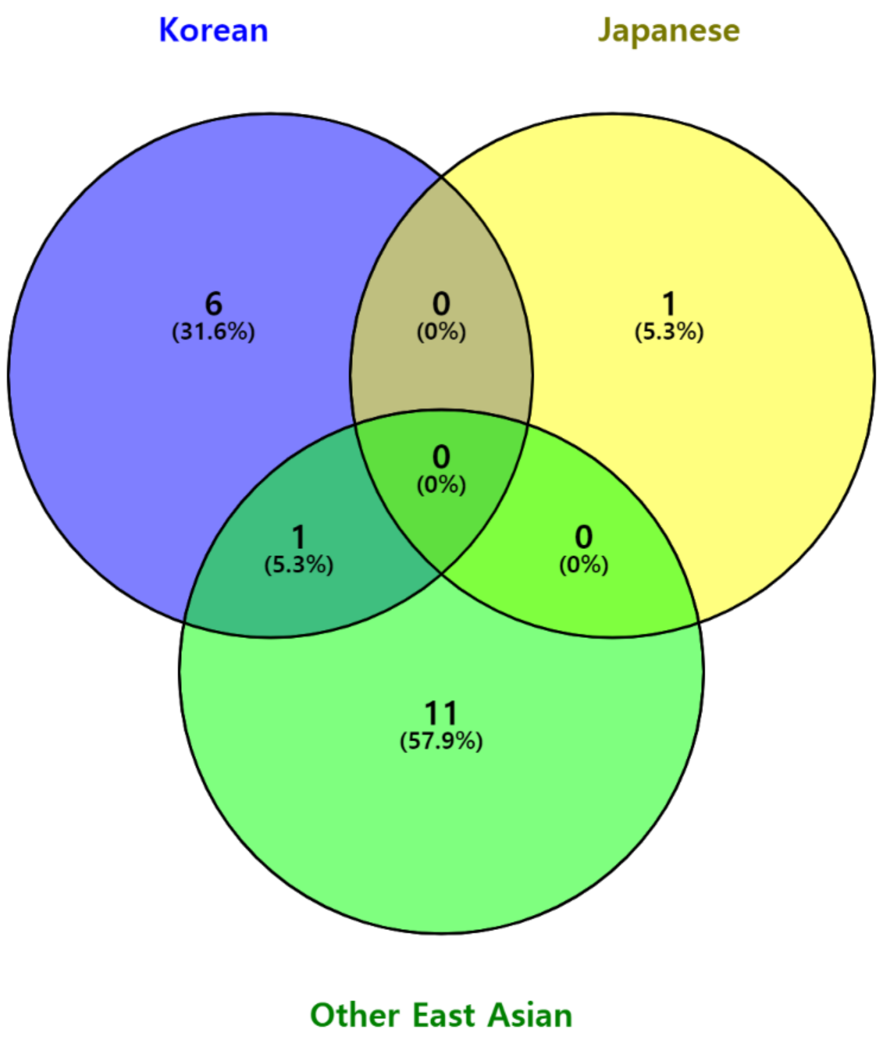

Supplementary Figure 1. Venn diagram of pathogenic variants and likely pathogenic variants of East Asians in gnomAD
